# Effects of N and P enrichment on plant photosynthetic traits in alpine steppe of the Qinghai-Tibetan Plateau

**DOI:** 10.1186/s12870-022-03781-9

**Published:** 2022-08-13

**Authors:** Hao Shen, Shikui Dong, Jiannan Xiao, Yangliu Zhi

**Affiliations:** 1 School of Grassland Science, Beijing Forestry University, Beijing, 100083 China; 2 School of Environment, State Key Joint Laboratory of Environmental Simulation and Pollution Control, Beijing Normal University, Beijing, 100875 China; 3 Department of Natural Resources, Cornell University, Ithaca, NY 14853 USA

**Keywords:** Qinghai-Tibetan plateau; alpine steppe, N and P enrichment, Photosynthesis, Biomass

## Abstract

**Background:**

N (nitrogen) and P (phosphorus) play important roles in plant growth and fitness, and both are the most important limiting factors that affect grassland structure and function. However, we still know little about plant physiological responses to N and P enrichment in alpine grassland of the Qinghai-Tibetan Plateau. In our experiment, five dominant common herbaceous species were selected and their photosynthetic parameters, leaf N content, and aboveground biomass were measured.

**Results:**

We found that species-specific responses to N and P enrichment were obvious at individual level. N addition (72 kg Nha^−1^ yr^−1^), P addition (36 kg Pha^−1^ yr^−1^) and NP addition (72 kg Nha^−1^ yr^−1^and 36 kg P ha^−1^ yr^−1^, simultaneously) significantly promoted net photosynthetic rate of *Leymus secalinus*. Differential responses also existed in the same functional groups. Responses of forb species to the nutrients addition varied, *Aconitum carmichaeli* was more sensitive to nutrients addition including N addition (72 kg Nha^−1^ yr^−1^), P addition (36 kg Pha^−1^ yr^−1^) and NP addition (72 kg Nha^−1^ yr^−1^and 36 kg P ha^−1^ yr^−1^). Responses of plant community photosynthetic traits were not so sensitive as those of plant individuals under N and P enrichment.

**Conclusions:**

Our findings highlighted that photosynthetic responses of alpine plants to N and P enrichment were species-specific. Grass species *Leymus secalinus* had a higher competitive advantage compared with other species under nutrient enrichment. Additionally, soil pH variation and nutrients imbalance induced by N and P enrichment is the main cause that affect photosynthetic traits of plant in alpine steppe of the Qinghai-Tibetan Plateau.

**Supplementary Information:**

The online version contains supplementary material available at 10.1186/s12870-022-03781-9.

## Introduction

Nitrogen (N) and phosphorus (P) are the most important components that affect plant growth and development in terrestrial ecosystem [1–3]. N and P play an important role in the synthesis of chlorophyll and photosynthetic enzymes [4]. Therefore, N and P fertilization would have a large influence on plant photosynthetic processes [5]. Previous studies have showed that moderate N and P addition can increase plant photosynthetic capacity, enhance grassland productivity and shift community composition [6, 7]. However, N and P enrichment can also cause negative effects, such as soil acidification, soil nutrients imbalance and thus to cause plant diversity loss [8].

Photosynthetic traits are comprehensive reflection of plant physiological status, which can measure the growth difference among different plants and the degree of environmental influence, and is closely related to plant growth and biomass accumulation. So far, no consistent results have been showed about how N and P enrichment affects plant photosynthetic traits for plant species-specific attribution. Nitrogen always has a close relationship with photosynthetic capacity, for the photosynthetic machinery and proteins related with Calvin cycle and thylakoids are mostly made-up of N element [9–11]. N addition can enhance plant net photosynthetic rate by supplying more N resource in N-limited ecosystem, however N enrichment usually negatively undermine plant net photosynthetic rate of some N-sensitive species by breaking soil nutrients balance [12]. P is the main component of chemical substances such as nucleic acids, ATP (adenosine-triphosphate) and phospholipids in the photosynthetic process, and it is also the most easily fixed and transformed element in soil [5]. Projected N addition may also aggravate P limitation in terrestrial ecosystem [12, 13], as excessive N input is usually thought to cause soil acidification and P leaching losses [14]. Moderate P addition can stimulate plant net photosynthetic rate by enhancing plant light use efficiency and stomatal conductance [15, 16]. Additionally, P supply may also modify the relationship between N and photosynthetic processes [17, 18]. At present, the studies on P addition effects mostly focused on the form, conversion, availability of soil P, and soil microbe dynamics [19–22]. Compared with N addition, P addition and their coupling effects on plant photosynthetic traits have been scarcely experimented in grassland ecosystems. Some studies found that N and P enrichment may also restrict plant photosynthetic rate by reducing leaf area and excessive nutrient input [23, 24].

In the alpine grasslands, responses to nutrient limitation may differ among species, this may be associated with the contrasting carbon and nutrient economies of different forms [25, 26], and interspecific eco-physiological adaptations disparity [27, 28]. There are also studies demonstrated that plants of different functional types show different photosynthetic capacity when the availability of N and P changes [29]. Generally, plant community shift could be predicted from individuals before ecosystem processes are largely influenced [30]. Eco-physiological responses of dominant species can reflect underlying mechanisms that lead changes in grassland community under N and P addition to some degree [28].

The objective of this study is to determine the impacts of N and P enrichment on plant and soil properties involved in N and P cycling in alpine steppe. Compared with N addition experiments, most experiments of P addition and NP addition on grassland ecosystem are concentrated on community level. The effects of N addition, P addition and their coupling effects on eco-physiological responses of alpine plants still remains unclear at both individual and community level [31]. Here, we conducted an experiment in an alpine steppe of the Qinghai-Tibetan Plateau to examine the eco-physiological responses of dominant plant species and predict the responses of whole plant community to N addition, P addition and their coupling effects. As alpine regions are usually N-limited [32] or being shift to P-limited [33], so we hypothesized that: (1) N and P enrichment may promote the photosynthetic capacity of plant and thus to promote productivity in alpine steppe. In addition, Some studies found that different species have different patterns of N and P allocation and nutrient economies [25, 26]. Divergent adaptation mechanisms of among species may be due to their biological characteristics [28]. Based on this, we hypothesized that: (2) plant photosynthetic responses to N and P enrichment might be species-specific.

## Material and methods

### Site description

**Fig. 1:**
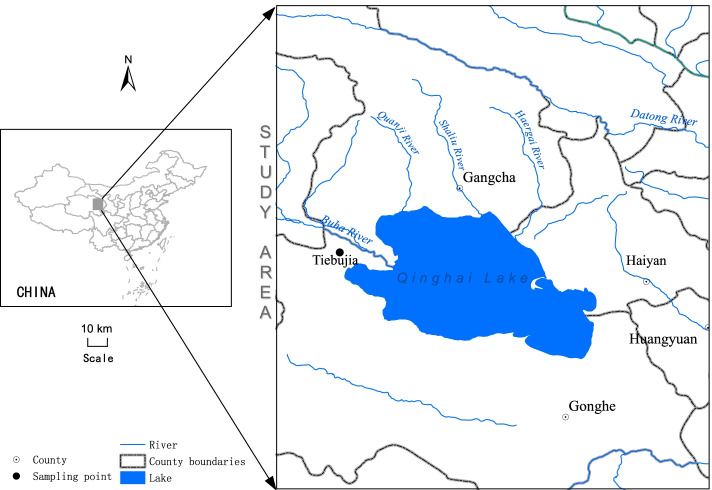
Location of the study site

The field experiment was carried out in an alpine steppe located at Tiebujia Town of Gonghe County (99°35′E, 37°02′N, 3270 m ASL) in Qinghai province, China (Fig. 1). The alpine steppe is with loam-clay soil. The mean annual temperature in alpine steppe is about 0 °C, the mean annual precipitation is about 377 mm, and the annual evaporation is about 1484 mm.

### Experimental design

In 2012, the grassland with an area of 20 m × 20 m was fenced with iron fence (1.2 m high). In 2018, four nutrient addition regimes were established in this area (randomized blocks design): control (CK, with neither N nor P addition); N addition (N, 72 kg Nha^−1^ yr^−1^); P addition (P, 36 kg P ha^−1^ yr^−1^); and combined N and P addition (NP, 72 kg Nha^−1^ yr^−1^and 36 kg P ha^−1^ yr^−1^, simultaneously). In alpine steppe, the N-saturated load is estimated by 40-50kgNha^−1^ yr^−1^ [34, 35], so the N addition rate largely simulated N critical load, while P addition rate was based on the requirement by the alpine plant communities [36]. Three plots in the fenced area (2 m by 5 m) were chosen as the replication of each treatment. All the plots in the treatments were similar in topographies and land use histories. Ammonium nitrate (NH_4_NO_3_) and calcium superphosphate (Ca(H_2_PO_4_)_2_) were applied as N fertilizer and P fertilizer form respectively in early May, July, and September each year since 2018. In this experiment, we selected five dominant species (according to their coverage) that exist in all plots to do the following parameters measurement (Table 1). *Leymus secalinus* is a perennial grass of Gramineae, with developed underground rhizomes and strong adaptability, and it is one of the main constructive and dominant species in the alpine grasslands on the Qinghai-Tibet Plateau. *Agropyron cristatum* is also a perennial grass of Gramineae, with well-developed fibrous roots and strong adaptability. *Aster tataricus*, *Potentilla multifida* and *Aconitum carmichaeli* are three perennial herbages belonging to Compositae, Rosaceae and Ranunculaceae, respectively. The five species have different morphological characteristics.

**Table 1:** List of selected species (dominant species) in this study site

Species	Family	Functional type	Life types	Photosynthetic type	Coverage
*Leymus secalinus*	Gramineae	Grass	Perennials	C3	50%
*Agropyron cristatum*	Gramineae	Grass	Perennials	C3	10%
*Aster tataricus*	Compositae	Forb	Perennials	C3	10%
*Potentilla multifida*	Rosaceae	Forb	Perennials	C3	10%
*Aconitum carmichaeli*	Ranunculaceae	Forb	Perennials	C3	5%

### Photosynthetic traits

In early August of 2019 (the peak growing period for alpine plants in this region), net photosynthetic rate (*P*_*N*_), transpiration rate (*T*_r_), stomatal conductance (*g*s), and intercellular CO_2_ concentration (*C*_i_) of each selected herbage species were measured using the Li-6800 (Li-Cor, Lincoln, NE, USA) with light availability of 1500 PAR between 9:00 and 12:00 am. The chamber CO_2_ concentration was maintained at 400 μmol·mol^−1^ with CO_2_ injection system, while leaf temperature was kept at 25 °C at a relative humidity between 60%-70%. Water use efficiency (WUE) was calculated as *P*_*N*_/*T*_r_. Five fully expanded leaves in the upper portion of each herbage species were selected for measurements. Also, five replicates were used for each species in this study.

### Sampling and measurement

The aboveground plant material of selected species were harvested and placed in sealed polyethylene bags and then dried at 70 ℃ for 48 h to constant weight. The dried materials were then ground to a fine powder with a vibrating sample mill (FW100, Tianjin Taisite Instrument Co., LTD, China) for subsequent analysis. Soil samples to the depth of 20 cm were collected by a 3.5 cm-diameter soil probe near the location of plant sampling. Then the soil samples were air-dried to constant weight and sieved through a 0.15-mm mesh. Plant and soil N content was measured by elemental analyzer (EA 3000, Italy). Soil NH_4_^+^-N and NO_3_^‐^-N content were measured using a flow injection auto‐analyzer (AACE, Germany). Soil Available phosphorus (AP) and available potassium (AK) was measured using an inductively coupled plasma spectrometer (SPECTRO ARCOS EOP, Germany). A glass electrode was used to measure soil pH in the supernatant by homogeneously mixing 5 g of soil and 25 ml of water [37].

### Data analysis

We calculated the mean of measured plant traits for each species in each plot. The relative abundance of each selected species was calculated. The community-weighted means (CWM) of measure plant traits were calculated using the following formula [38]:

$$\mathrm{CWM}\;=\;\sum_{i=1}^n\;{\mathrm p}_{\mathit i}\;{\mathrm s}_i$$

where p_*i*_ is the relative abundance of species *i*, and s_*i*_ is the mean value of plant traits in each treatment.

In addition, response ratio (RR) for each observation was calculated as the natural log of the response ratio RR = ln (*X*_*t*_/*X*_*c*_), where *Xt* is the mean of plant traits for each treatment and *X*_*c*_ is the mean of plant traits in associated unfertilized control [39]. More specifically, the mean, standard deviation (S) or standard error, and sample size for each observation were calculated to calculate the *RR*. The statistical analyses were performed using the software package R (4.0.3). Then, we used one-way ANOVA in SPSS 22.0 software (SPSS Inc) to estimate the effect of nutrients addition on all plant traits. Thereafter, the least square difference (LSD) tests were used to conduct post hoc mean comparisons of each plant traits of each species under different treatments. Additionally, in order to visualize the relationship among all plant and soil variables, a correlation matrix diagram and a PCA analysis were successfully developed in R.

## Results

### Effects of N and P addition on photosynthetic capacity of five dominant common plant species

**Fig.2:**
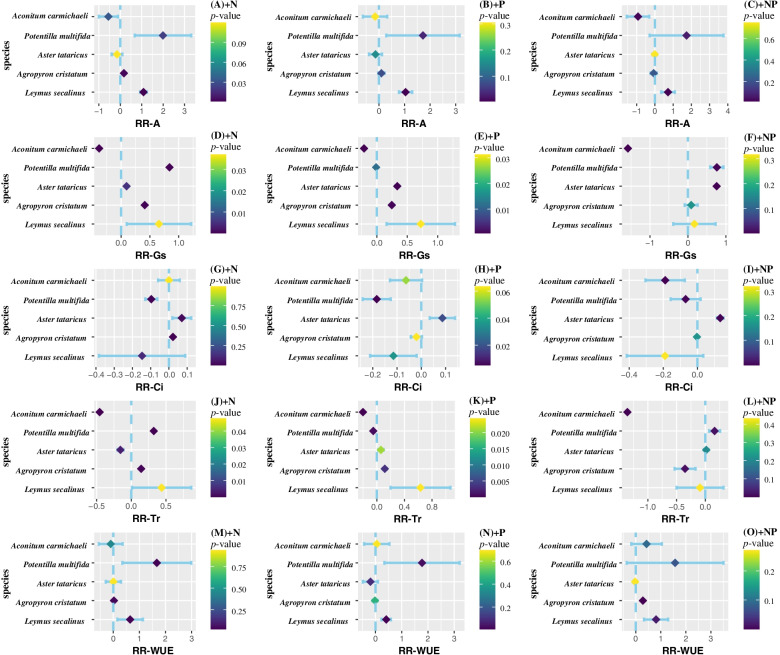
Photosynthetic parameters from five dominant common plant species (grasses and forbs) in the nutrient fertilization experiment fertilized with nitrogen (N), phosphorus (P) and both (NP). Panel (A), (B), (C) show response ratio of net photosynthetic rate (*A*) under N, P and both NP fertilization respectively. Panel (D), (E), (F) show response ratio of stomatal conductance (*Gs*) under N, P and both NP fertilization respectively. Panel (G), (H), (I) show response ratio of intercellular CO_2_ concentration (*Ci*) under N, P and both NP fertilization respectively. Panel (J), (K), (L) show response ratio of transpiration rate (*Tr*) under N, P and both NP fertilization respectively. Panel (M), (N), (O) show response ratio of water use efficiency (*WUE*) under N, P and both NP fertilization respectively. ***RR***: response ratio

Species-specific responses to nutrient additions were obvious (Fig. 2). N addition, P addition and NP addition significantly promoted net photosynthetic rate of *Leymus secalinus* (*p* < 0.05). The net photosynthetic rate of *Agropyron cristatum* and *Aster tataricus* showed no significant responses to N and P addition. Single N and P addition significantly promoted the net photosynthetic rate of *Potentilla multifidi* (*p* < *0.05*). N and NP addition significantly decreased the net photosynthetic rate of *Aconitum carmichaeli* (*p* < *0.05*), while P addition had no significant effects. All nutrient addition treatments significantly decreased the *Gs* of *Aconitum carmichaeli* (*p* < 0.05). Single N and P addition significantly promoted the *Gs* of *Leymus secalinus*, *Agropyron cristatum* and *Aster tataricus* (*p* < *0.05*). All of nutrient addition treatments significantly increased the *Ci* of *Aster tataricus* (*p* < 0.05). Single N and P addition significantly increased the *Tr* of two grass species (*p* < 0.05). *Leymus secalinus* kept a significant higher *WUE* under all treatments of nutrient additions compared with other herbaceous species.

### Effects of N and P addition on photosynthetic characteristics, N content, height and AGB of the whole plant community

**Fig. 3:**
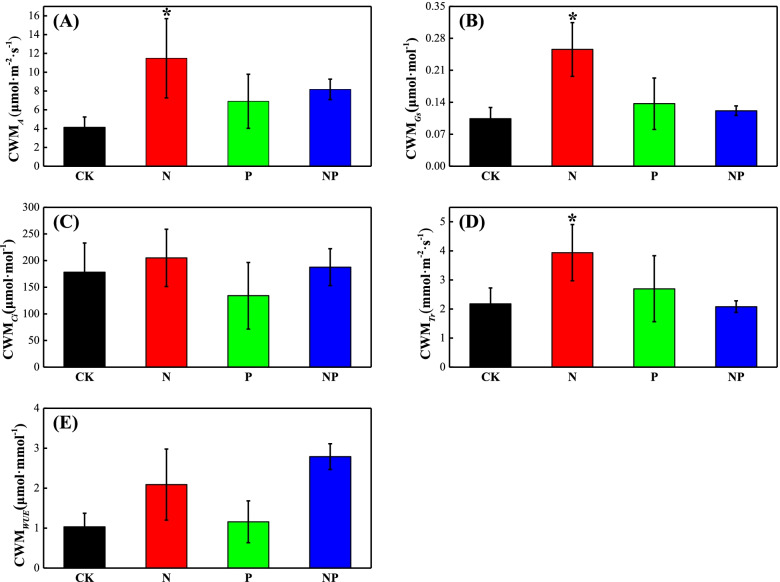
Effects of N and P fertilization on community weighed mean (CWM) of photosynthetic parameters. **(A)** *A*: net photosynthetic rate, **(B)** *Gs*: stomatal conductance, **(C)** *Ci*: intercellular CO_2_ concentration, **(D)** *Tr*: transpiration rate, **(E)** *WUE*: water use efficiency.CK: control, N: N fertilization, P: P fertilization, NP: N plus P fertilization.* indicates significant difference between treatments (*p* < 0.05)

**Fig. 4:**
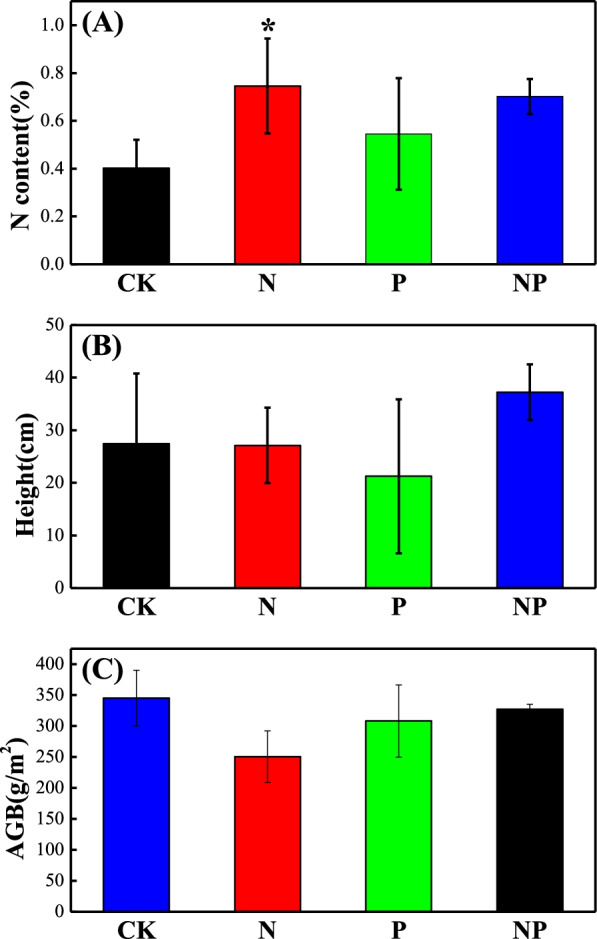
Effects of N and P fertilization on total aboveground biomass (AGB) and community weighed mean (CWM) of N content and height. CK: control, N: N fertilization, P: P fertilization, NP: N plus P fertilization.* indicates significant difference between treatments (*p* < 0.05). **(A)** community N content, **(B)** *community height*, **(C)**community aboveground biomass

N addition significantly promoted the *A, Gs* and *Tr* of the whole plant community (*p* < 0.05) (Fig. 3), but did not significantly affect the community *Ci* and *WUE* of the plant community. Both P and NP addition had no significant effects on photosynthetic capacity of the plant community (*p* > 0.05). Only N addition significantly increased N content of the community(*p* < 0.05, Fig. 4A), and N addition, P addition and their combination had no significant effects on the height and AGB of the whole community (Fig. 4B and C).

### Relationship among plant eco-physiological traits and soil properties

**Fig. 5:**
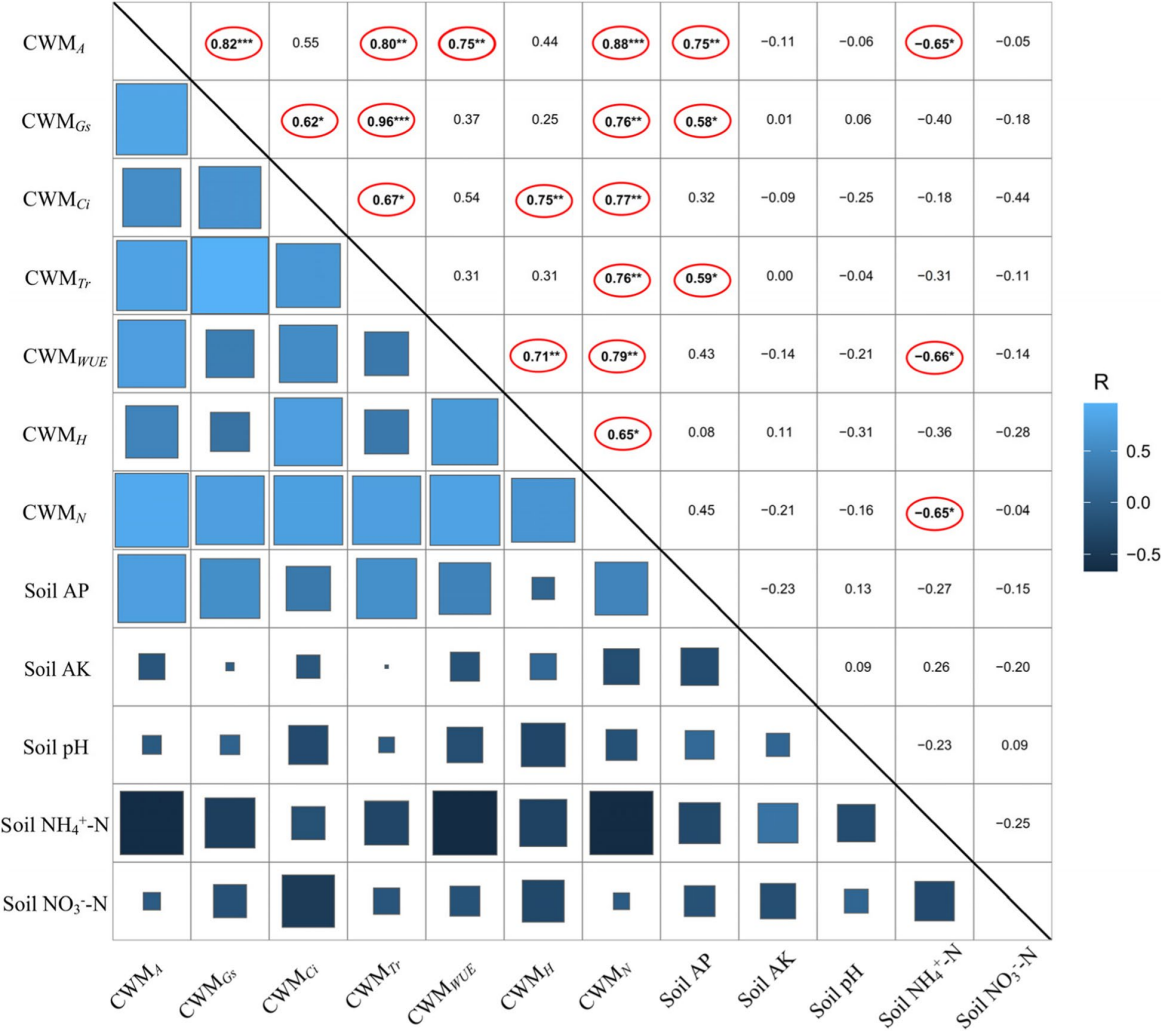
Correlation of community weighed mean (CWM) values among all plant eco-physiological traits and soil nutrients. *A*: net photosynthetic rate, *Gs*: stomatal conductance, *Ci*: intercellular CO_2_ concentration, *Tr*: transpiration rate, *WUE*: water use efficiency.CK: control, N: N fertilization, P: P fertilization, NP: N plus P fertilization.* indicates significant difference at the level of *p* < 0.05,** indicates significant difference at the level of *p* < 0.01, ***indicates significant difference at the level of *p* < 0.001.Soil AP: Soil available phosphorus, Soil AK: Soil available potassium, Soil NH_4_^+^-N: Soil ammonium nitrogen, Soil NO_3_^−^-N: Soil nitrate nitrogen

**Fig. 6:**
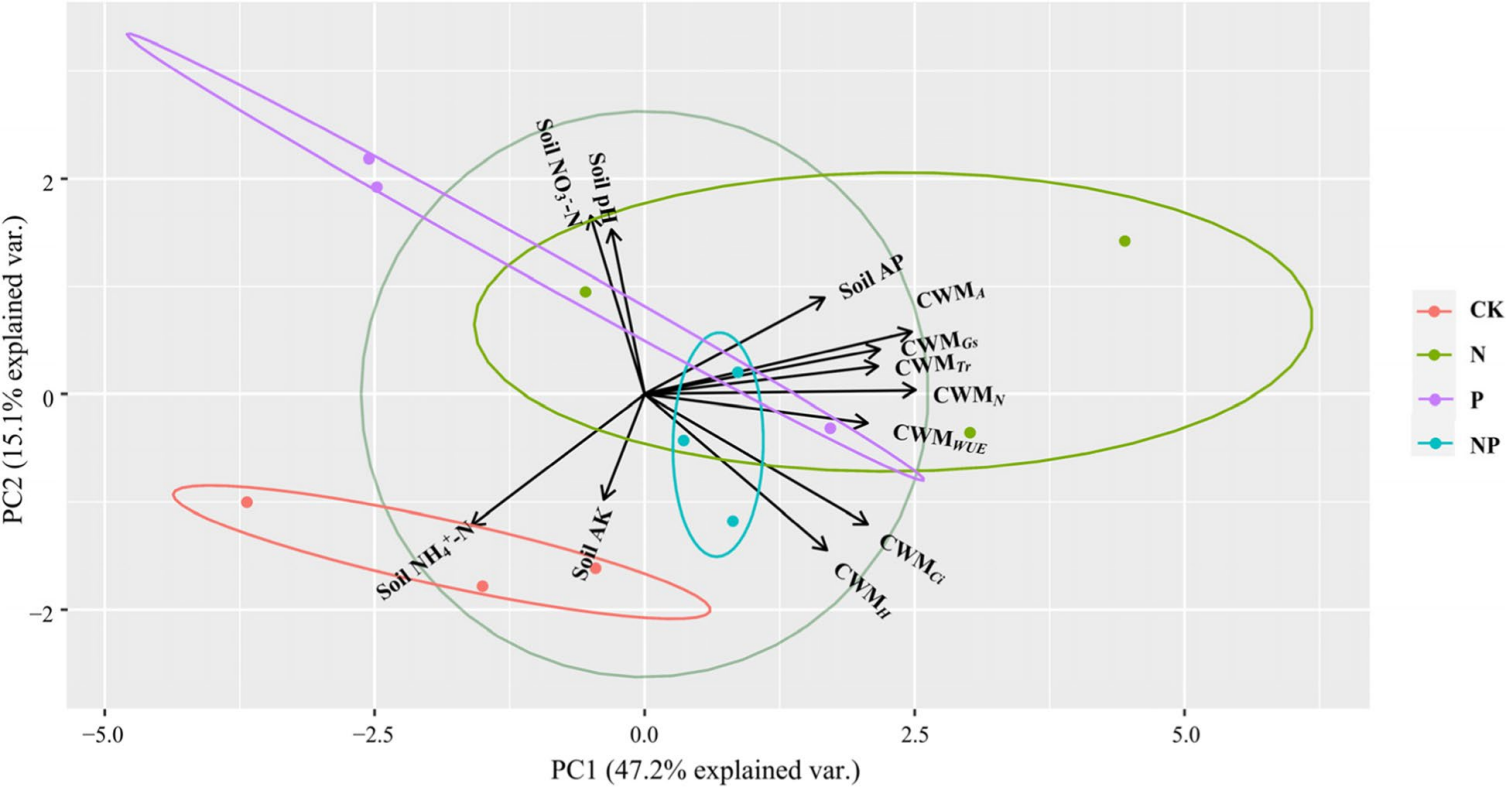
Principal component analysis (PCA) of plant and soil variables. The smaller angle between two variable arrows indicates stronger correlation, as the cosine of the angle between variable arrows equals their correlation coefficients. The length of vector arrows indicate weight of the variables. *A*: net photosynthetic rate, *Gs*: stomatal conductance, *Ci*: intercellular CO_2_ concentration, *Tr*: transpiration rate, *WUE*: water use efficiency.CK: control, N: N fertilization, P: P fertilization, NP: N plus P fertilization. Soil AP: Soil available phosphorus, Soil AK: Soil available potassium, Soil NH_4_^+^-N: Soil ammonium nitrogen, Soil NO_3_^−^-N: Soil nitrate nitrogen

CWM_*A*_ was positively related with CWM_*Gs*_ (*r* = 0.82, *p* < 0.01), CWM_*Tr*_ (*r* = 0.80, *p* < 0.01), CWM_*WUE*_ (*r* = 0.75, *p* < 0.01), CWM_*N*_ (*r* = 0.88, p < 0.001), soil NH_4_^+^-N (*r* = 0.75, *p* < 0.01) and soil AP (*r* = -0.65, *p* < 0.05) (Fig. 5). PCA showed that PC1 and PC2 explained 62.3% of the variance of all plant and soil variables (Fig. 6). Although N, P and NP addition presented a clear separation with CK, no clear separation was found under N, P and NP addition treatments. Soil NH_4_^+^-N and soil AP presented the largest weight in all measured soil properties, while CWM_*A*_ accounted for the largest weight in all measured plant traits.

## Discussion

Alpine regions are typically thought to be N-limited [32, 40] or being shifted to P-limited [33]. Cold temperatures, short growing seasons and low nutrient supply are usually the limiting factors of alpine grassland productivity [41, 42]. Generally, long-term moderate N and P supply in grassland can increase the biomass, leaf area, or shift species composition [43]. Although N and P addition can alleviate nutrient deficiency to some degree, such effects seem to be not so obvious in short term at the community level. However, N and P enrichment can obviously affect growth and photosynthesis of plant individual through the fertilization-induced soil nutrients imbalance and soil pH variation. In this study, soil acidification was not obvious under N and P addition, yet soil nutrients tended to fluctuate with nutrient addition (Table S1). And this suggested that soil nutrients balance is much more sensitive than soil pH, while hysteresis effect might exist for a short term nutrient addition experiment. In addition, soil AP and soil NH_4_^+^-N are the two most important factors that affect plant community traits in this study. Soil AP has a closely positive effects with plant community traits while soil NH_4_^+^-N has an obvious negative effects. This suggested that plants showed a more demand of P under nutrients enrichment, while N addition rate in this study is a critical load [35].

The species-specific responses to N and P addition may depend largely on their eco-physiological adaptation in the plant community. Generally, compared with forbs and other functional types, grass can be much more competitive under nutrient addition for its higher nutrient and light use efficiency as well as high nutrient critical load [44–46]. The responsive variations also appeared in the same functional groups, i.e., grass species *Leymus secalinus* showed improved photosynthetic capacity under nutrients addition, while *Agropyron cristatum* tended to be non-responsive to N and P addition, implying that *Leymus secalinus* may possess a relatively higher nutrients use efficiency [47, 48]. This may also suggest that interspecific competition for resources existed in the same functional groups, and the ability for nutrients uptake varied among different species. Although previous studies have reported that N addition had no significant effect on forb [49], we found that the net photosynthetic rate of forb species (*Aconitum carmichaeli*) was obviously negatively affected by N and NP addition in this study. *Aster tataricus* showed non-responsive to nutrients addition, suggesting its high inner stability to exogenous nutrients input. Both single N and P addition promoted the photosynthetic capacity of *Potentilla multifidim* while NP addition had no significant effect. This may be because N and P coupling addition exceed the nutrients requirement of *Potentilla multifidim,* thus higher nutrients load might offset the positive effects brought by N and P addition. Overall, the different responses of plant photosynthetic capacity to N and P addition suggested different adaptation mechanism of these species. Although previous studies stated that the plants within the same functional group may persist similar responses to external environmental changes [50], species-specific responses in our study suggested individual disparity of the plants even from the same functional groups. Generally, moderate P addition could alleviate detrimental effects induced by excessive N input [51]. However, *Agropyron cristatum* and *Aster tataricus* were non-responsive to N and P addition in our study, this may be because high intraspecific and interspecific competition as well as low nutrient utilization efficiency of these species. N and P addition had no negative effects on grass species, indicating that grass species may possess a higher nutrients use efficiency or nutrient critical load than other species [52].

At the community level, significant photosynthetic response was only found in N addition treatment, suggesting the photosynthetic capacity of the whole community was still being limited by N resource. Despite obvious increase in plant N uptake of all species, some of the species photosynthetic capacity was not elevated in this study, implying that excessive nutrient uptake might have not partitioned to photosynthetic components with more nutrient supply [53]. In our study, photosynthetic capacity of the dominant common grass species, in contrast to forb species, had a much higher N critical load, suggesting that grasses can better adapt to high nutrients supply than forbs. In addition, N and P addition increased the net photosynthetic rate of some species via the increase of stomatal conductance, suggesting the close relations between photosynthetic capacity and stomatal behavior [54]. Higher stomatal conductance can increase CO_2_ supply to intercellular space for plant photosynthesis [55]. Nutrients addition could improve plant photosynthetic capacity by enlarging cell size and making the cell wall thinner to enhance stomatal conductance [56]. The variation of the net photosynthetic rate was inconsistent with that of stomatal conductance for some species, indicating that non-stomatal limitation (chlorophyll and carboxylation) may play an important role under N and P enrichment [28, 57, 58]. Overall, the different photosynthetic responses among the plant species in the alpine steppe suggested that long-term projected N and P addition may have the potential to change plant species composition and finally lead to the change of grassland community structure and function.

Alpine grassland productivity is usually limited by N and P supply [12]. However, the effects of N and P addition on grassland productivity is still inconsistent [59–61]. Some studies have showed strong effects of fertilization on plant productivity [62–64]. Positive effects of N and P addition on grassland community productivity may depend on various factors, such as nutrient addition rate [62] and precipitation [65]. We didn’t see obviously responses of plant productivity at the community level to N and P addition, indicating that grassland community productivity can remain much stable and non-responsive to short-term nutrient addition in alpine regions. This result may also be associated with the nutrient loss in the fertilization process induced by rainfall or other environmental factors [66].

Overall, we found that not all of the photosynthetic capacity were promoted by the N and P enrichment and the productivity was also not obviously promoted. Such result is inconsistent with our first hypothesis. This suggested that some species might be negatively sensitive to nutrient enrichment, through alpine grassland ecosystems are usually both N and P limited [67, 68]. However, we found that plant photosynthetic responses to N and P enrichment were indeed differential. This complied with our second hypothesis that plant photosynthetic responses to N and P enrichment are species-specific. Soil nutrients dynamics are important influencing factors for plant photosynthetic traits [69]. Soil properties change induced by N and P enrichment would influence plant photosynthesis, affect plant fitness and grassland productivity, and finally alter ecosystem functioning [12, 66, 70–72]. On the whole, different species have different patterns of N and P allocation to various components under N and P enrichment, and such differences can finally cause the disparity in photosynthetic traits among species.

## Conclusion

Our study highlights that the plant photosynthetic responses to N and P addition are species-specific. *Leymus secalinus* has an absolute superiority of photosynthetic capacity under higher N and P supply. Not all forb species are sensitive in photosynthetic responses to higher N and P addition. Responses of plant community functional traits were not so sensitive as those of plant individuals. In the future, a long-term N and P fertilization with multi-level still should be applied to examine the photosynthetic traits variation of different species and community vegetation dynamics to optimize fertilization effects in alpine regions.

## Supplementary Information

**Additional file 1: Table S1**. Soil pH and available nutrients variation under N and P addition.

## Data Availability

All data generated or analyzed during this study are included in this article.

## Abbreviations

*A*: Net photosynthetic rate
*Gs*: Stomatal conductance
*Ci*: Intercellular CO_2_ concentration
*Tr*: Transpiration rate
*WUE*: Water use efficiency
CK: Control
N: N fertilization
P: P fertilization
NP: N plus P fertilization.
Soil AP: Soil available phosphorus
Soil AK: Soil available potassium
Soil NH_4_^+^-N: Soil ammonium nitrogen
Soil NO_3_^−^-N: Soil nitrate nitrogen
CWM: Community-weighted means
