# Supplementary material for: Effects of N and P enrichment on plant photosynthetic traits in alpine steppe of the Qinghai-Tibetan Plateau
Source: BMC Plant Biol. 2022 Aug 13;22:396. doi: 10.1186/s12870-022-03781-9 (PMC9375904; doi:10.1186/s12870-022-03781-9)
Supplement: Supplementary file 1 — Additional file 1: Table S1. Soil pH and available nutrients variation under N and P addition. [file 12870_2022_3781_MOESM1_ESM.docx]

|  | AK(mg/kg) | AP(mg/kg) | NH_4_^+^-N(g/kg) | NO_3_^-^-N(g/kg) | pH |
| --- | --- | --- | --- | --- | --- |
| CK | 100.4332±13.08455 | 0.015±0.00201 | 0.1308±0.02858 | 0.0713±0.0177 | 7.5633±0.00577 |
| N | 91.068±20.93202 | 0.0188±0.00623 | 0.0529±0.01102 | 0.0965±0.00897 | 7.54±0.03464 |
| P | 88.5113±5.09595 | 0.0157±0.00073 | 0.0682±0.02974 | 0.2216±0.05495 | 7.5533±0.02887 |
| NP | 85.6722±34.16428 | 0.0139±0.00094 | 0.0444±0.01762 | 0.1284±0.01273 | 7.54±0.04359 |

**Table S1**. Soil pH and available nutrients variation under N and P addition.
